# Supplementary material for: Evaluation of Spectrum-Aided Visual Enhancer (SAVE) in Esophageal Cancer Detection Using YOLO Frameworks
Source: Diagnostics (Basel). 2024 May 29;14(11):1129. doi: 10.3390/diagnostics14111129 (PMC11171540; doi:10.3390/diagnostics14111129)
Supplement: Supplementary file 1 [file diagnostics-14-01129-s001.zip › diagnostics-2964049-supplementary.pdf]

*Article*

# Evaluation of Spectrum-Aided Visual Enhancer (SAVE) in Esophageal Cancer Detection: Supplementary Material

**Chu-Kuang Chou [1,2,3], Riya Karmakar [4], Yu-Ming Tsao [4], Lim Wei Jie [5], Arvind Mukundan [4], Chien-Wei Huang [6,7], Tsung-Hsien Chen [8], Chau-Yuan Ko [6,*] and Hsiang-Chen Wang [4,9,10,*]**

1 Division of Gastroenterology and Hepatology, Department of Internal Medicine, Ditmanson Medical Foundation Chia-Yi Christian Hospital, Chia-Yi 60002, Taiwan; vacinu@gmail.com
2 Obesity Center, Ditmanson Medical Foundation Chia-Yi Christian Hospital, Chia-Yi 60002, Taiwan
3 Department of Medical Quality, Ditmanson Medical Foundation Chia-Yi Christian Hospital, Chiayi 60002, Taiwan
4 Department of Mechanical Engineering, National Chung Cheng University, Chia-Yi 62102, Taiwan; karmakarriya345@gmail.com (R.K.); d09420002@ccu.edu.tw (Y.-M.T.); d09420003@ccu.edu.tw (A.M.)
5 Department of Computer Science, Multimedia University (Cyberjaya), Persiaran Multimedia, 63100 Cyberjaya, Malaysia; jaylim.wjie@gmail.com
6 Department of Gastroenterology, Kaohsiung Armed Forces General Hospital, 2, Zhongzheng 1st. Rd., Lingya District, Kaohsiung City 80284, Taiwan; forevershiningfy@yahoo.com.tw
7 Department of Nursing, Tajen University, 20, Weixin Rd., Yanpu Township, Pingtung County 90741, Taiwan
8 Department of Internal Medicine, Ditmanson Medical Foundation Chia-Yi Christian Hospital, Chiayi 60002, Taiwan; cych13794@gmail.com
9 Department of Medical Research, Dalin Tzu Chi Hospital, Buddhist Tzu Chi Medical Foundation, No. 2, Minsheng Road, Dalin, Chia-Yi 62247, Taiwan
10 Director of Technology Development, Hitspectra Intelligent Technology Co., Ltd., Kaohsiung City 80661, Taiwan
* Correspondence: gastroenterokjy@gmail.com (C.-Y.K.); hcwang@ccu.edu.tw (H.-C.W.)

**Abstract:** This document provides the supplementary material for the article "Utilizing Hyperspectral Imaging and YOLO Framework for Early Esophageal Cancer Detection". The first section explains about the YOLOv5 and YOLOv8 Architecture and the second section describes the definition of the object detection indicators used in this study.

**Keywords:** Esophageal Cancer, hyperspectral imaging, SAVE, dysplasia, YOLOv5, YOLOv8, narrowband imaging, white light imaging.

## S1. Yolo Architecture

### *S1.1 YOLOv5*

The YOLOv5 architecture adopts a single-stage detector design, featuring three integral components: Backbone, Neck, and Head, each playing a crucial role in the process of effective object detection. Figure S1 visually displays the YOLOv5 architecture [1]. Serving as the initial stage, the Backbone, named CSP-Darknet53, is responsible for extracting intricate feature representations from input images. This step ensures that the model captures nuanced details essential for precise object detection.

Incorporating both SPP (Spatial Pyramid Pooling) and PANet (Path Aggregation Network), the Neck component of YOLOv5 enhances the architecture's adaptability. SPP facilitates the extraction of feature pyramids, allowing the model to generalize effectively across objects of varying sizes and scales. Concurrently, PANet contributes to improved feature representation by aggregating information from different network paths. Together, these components empower YOLOv5 with the flexibility required for detecting objects across diverse scenarios.

The final component, the Head, marks the culmination of the architecture, where anchor boxes are applied to feature maps generated by the Neck. These anchor boxes serve as reference points for predicting the ultimate output, encompassing class labels, objectness scores, and bounding box coordinates. The strategic use of anchor boxes facilitates precise object localization and robust classification, underscoring YOLOv5's overall accuracy in object detection.

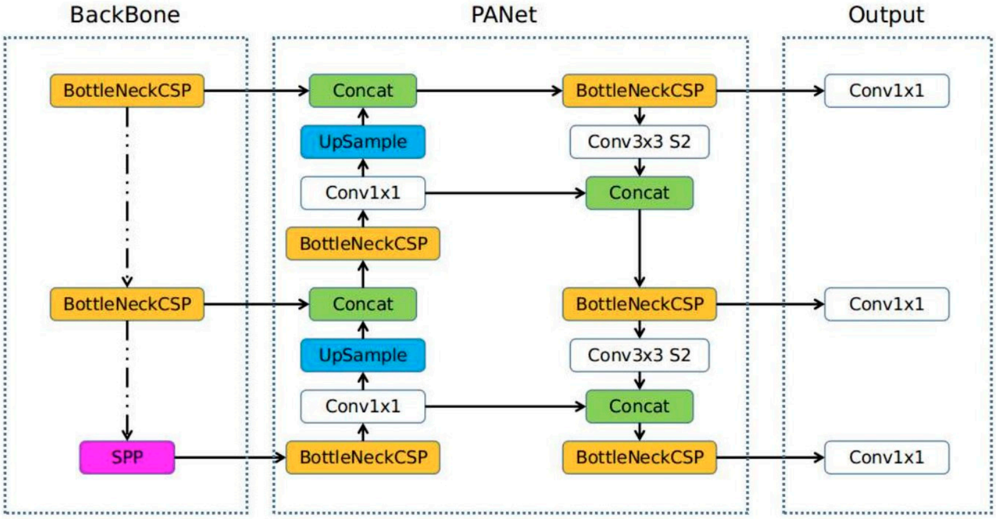


**Figure S1.** YOLOv5 Architecture [1]

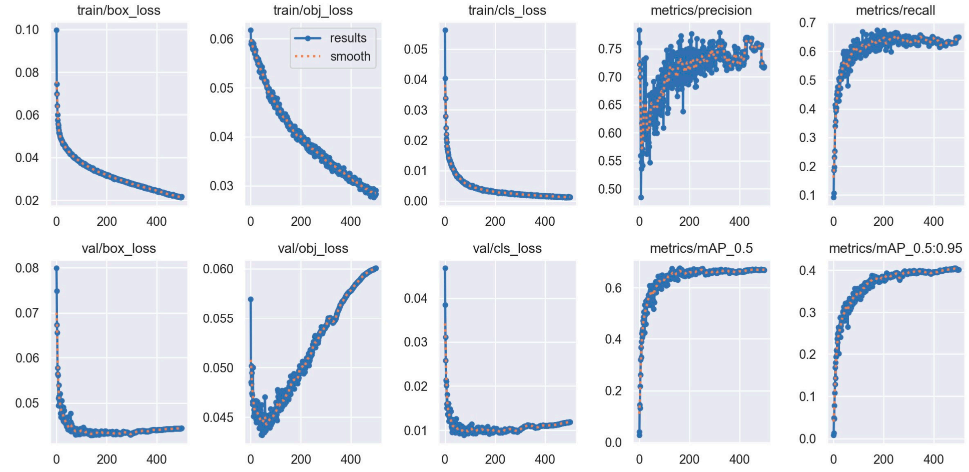


**Figure S2.** YOLOv5 White-Light image training set and validation set loss functions and convergence of precision, recall, and mean precision

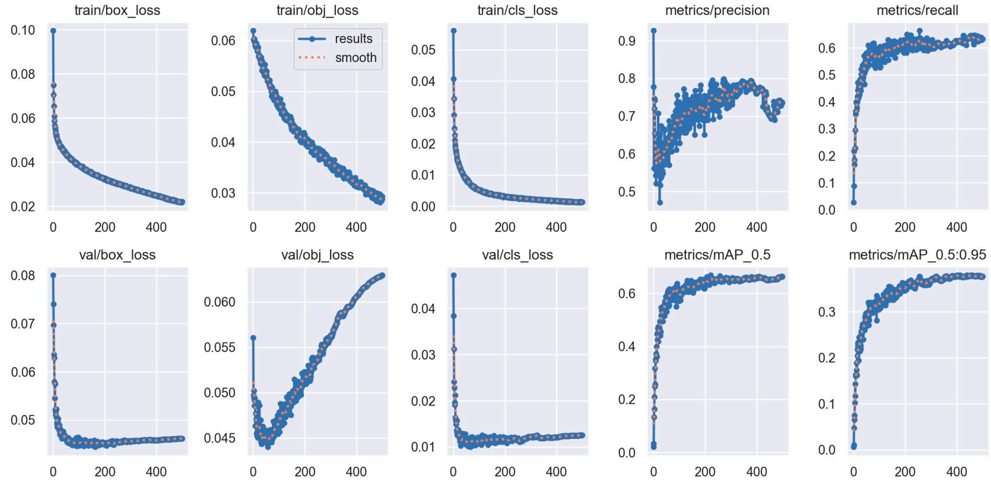


**Figure S3.** YOLOv5 Hyperspectral image training set and validation set loss functions and convergence of precision, recall, and mean precision

*S1.2 YOLOV8*

As per the official documentation, YOLOv8 stands as the most recent iteration of the YOLO object detection and image segmentation model created by Ultralytics. Representing a state-of-the-art model, YOLOv8 builds upon the accomplishments of its predecessors, introducing novel features and enhancements to elevate both performance and

adaptability. YOLOv8 architecture introduces several key enhancements, including mosaic data augmentation, anchor-free detection, a C2f module in the backbone, a decoupled head, and a modified loss function. Mosaic data augmentation combines four images to provide contextual information, optimizing model training. Anchor-free detection replaces predefined anchors, improving generalization and expediting Non-max Suppression. The C2f module in the backbone concatenates bottleneck module outputs, enhancing computational efficiency. The decoupled head separates classification and regression tasks, potentially causing misalignment. To address this, a task alignment score is introduced, guiding the model in selecting and optimizing positive samples using BCE, CIoU, and DFL loss functions. BCE ensures label prediction accuracy, CIoU refines bounding box positioning, and DFL optimizes boundary distribution for improved overall performance. Figure S4 illustrates the YOLOv8 architecture, highlighting its features for enhanced object detection [2].

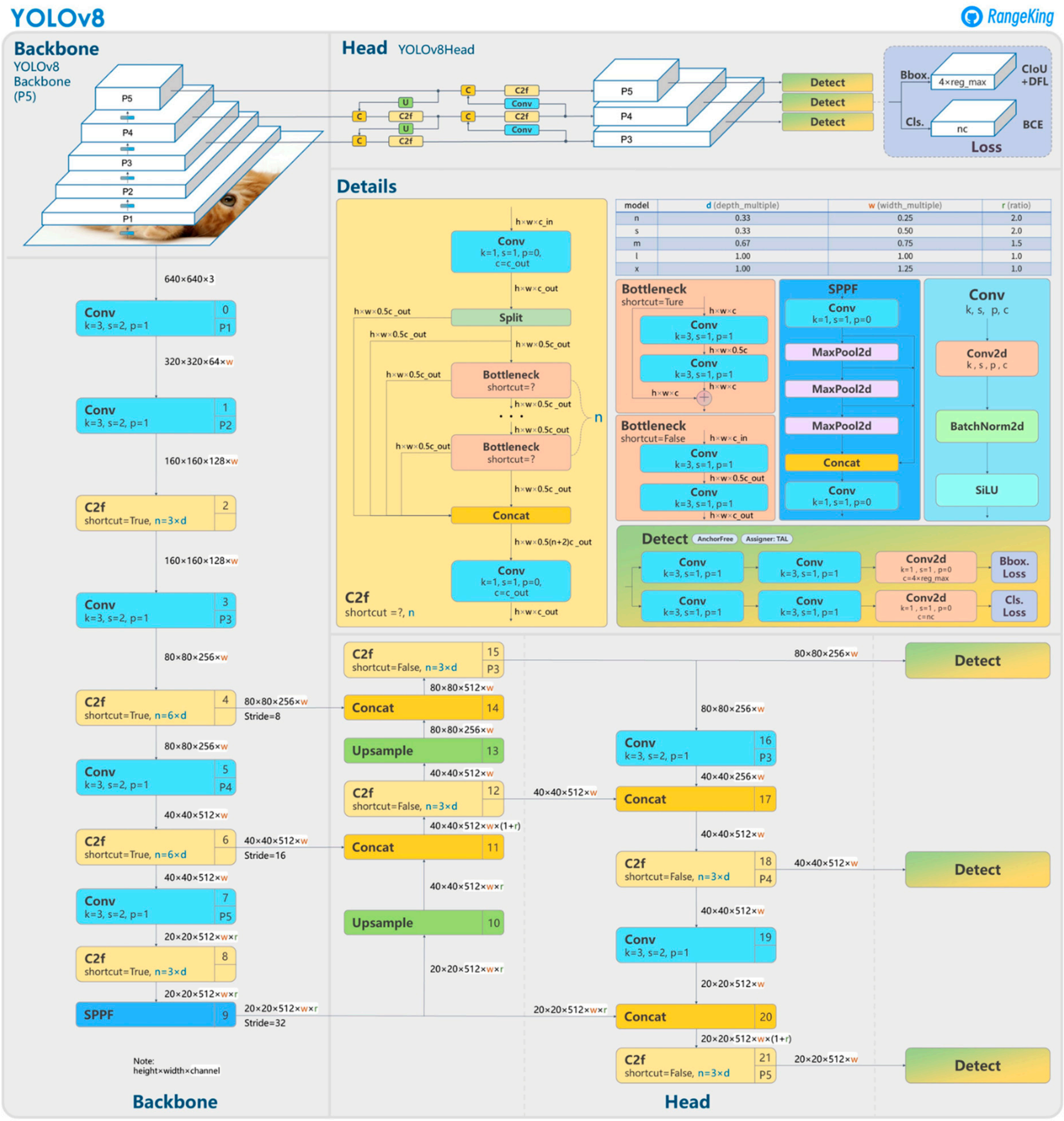


**Figure** S4. YOLOv8 Architecture [2]

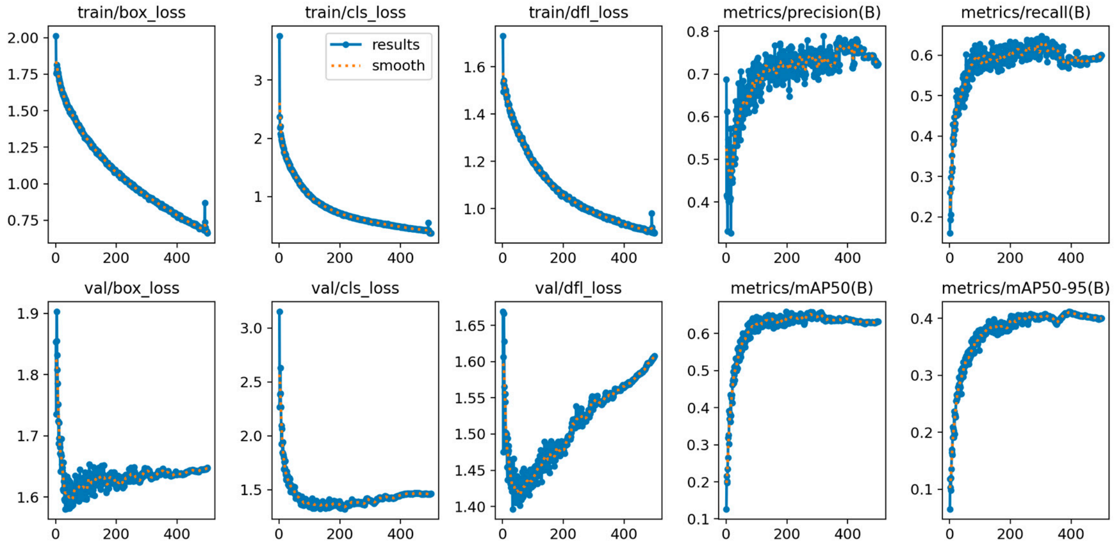


**Figure** S5. YOLOv8 White-Light image training set and validation set loss functions and convergence of precision, recall, and mean precision

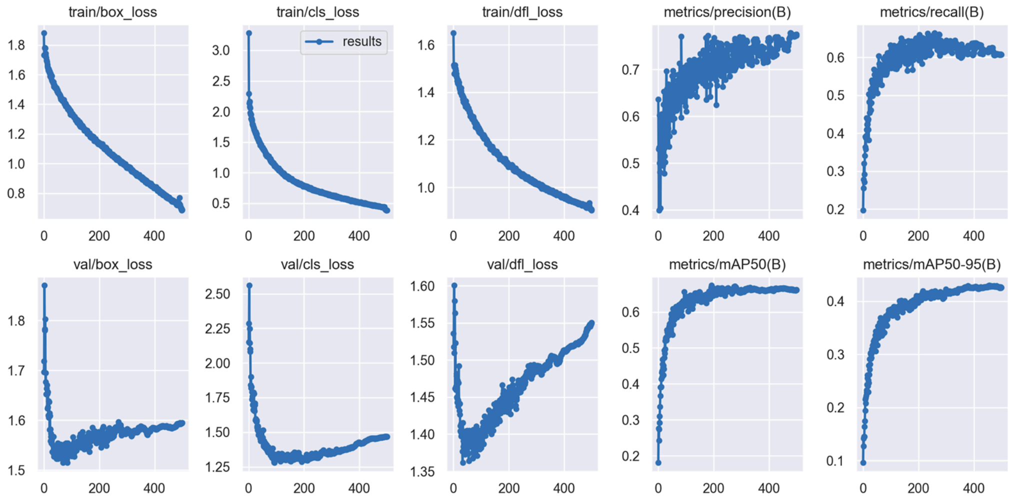


**Figure** S6. YOLOv8 Hyperspectral image training set and validation set loss functions and convergence of precision, recall, and mean precision

**S2. Equation of Object Detection Indicators**

In esophageal cancer detection, precision, recall, F1-score, mean Average Precision (mAP) and confusion metrics are key metrics for evaluating tailored object detection models [3]. Precision emphasizes the accuracy of positive predictions, ensuring alignment with actual symptoms. High precision implies reliable identification of true positives while minimizing false positives in esophageal cancer symptoms.

$$Precision = \mathrm{TP}/\,(TP + FP) \quad \text{(S1)}$$

Recall is crucial, measuring the model's sensitivity to capture all relevant cancer symptoms. High recall indicates effective identification, minimizing oversight.

$$Recall = \mathrm{TP}/\,(TP + FN) \quad \text{(S2)}$$

The F1-score, a harmonic mean of precision and recall, is vital, offering a balanced assessment of overall accuracy in esophageal cancer detection.

$$F1\text{-}Score = (2 \times \mathrm{Precision} \times \mathrm{Recall})/\,(\mathrm{Precision} + \mathrm{Recall}) \quad \text{(S3)}$$

mAP (mean Average Precision), specialized for esophageal cancer, considers precision-recall curves for each symptom, providing nuanced evaluation across manifestations. This metric calculates the average precision ($AP_k$) for each

class (k), where n represents the number of classes. The average precision of each class is then averaged to compute the mean Average Precision.

$$mAP = \frac{1}{n} \sum_{k=1}^{k=n} AP_k \qquad \text{(S4)}$$

**References**


1. seekFire, "Overview of model structure about YOLOv5 · Issue #280 · ultralytics/yolov5," .
2. R. King, "Brief summary of YOLOv8 model structure · Issue #189 · ultralytics/ultralytics," .
3. R. Khandelwal, "Evaluating performance of an object detection model," (2020).
